# Potential Association of Osteoporosis and Not Osteoporotic Fractures in Patients with Gout: A Longitudinal Follow-Up Study

**DOI:** 10.3390/nu15010134

**Published:** 2022-12-28

**Authors:** Mi Jung Kwon, Jae Yong Park, Sung Gyun Kim, Jwa-Kyung Kim, Hyun Lim, Joo-Hee Kim, Ji Hee Kim, Seong-Jin Cho, Eun Sook Nam, Ha Young Park, Nan Young Kim, Ho Suk Kang

**Affiliations:** 1 Department of Pathology, Hallym University Sacred Heart Hospital, Hallym University College of Medicine, Anyang 14068, Republic of Korea; 2 Department of Orthopedic Surgery, Hallym University Sacred Heart Hospital, Hallym University College of Medicine, Anyang 14068, Republic of Korea; 3 Department of Internal Medicine, Division of Nephrology, Hallym University Sacred Heart Hospital, Hallym University College of Medicine, Anyang 14068, Republic of Korea; 4 Department of Internal Medicine, Division of Gastroenterology, Hallym University Sacred Heart Hospital, Hallym University College of Medicine, Anyang 14068, Republic of Korea; 5 Department of Medicine, Division of Pulmonary, Allergy, and Critical Care Medicine, Hallym University Sacred Heart Hospital, Hallym University College of Medicine, Anyang 14068, Republic of Korea; 6 Department of Neurosurgery, Hallym University Sacred Heart Hospital, Hallym University College of Medicine, Anyang 14068, Republic of Korea; 7 Department of Pathology, Kangdong Sacred Heart Hospital, Hallym University College of Medicine, Seoul 05355, Republic of Korea; 8 Department of Pathology, Busan Paik Hospital, Inje University College of Medicine, Busan 47392, Republic of Korea; 9 Hallym Institute of Translational Genomics and Bioinformatics, Hallym University Medical Center, Anyang 14068, Republic of Korea

**Keywords:** gout, osteoporosis, osteoporotic fracture, risk factor, nationwide health care data, longitudinal follow-up study

## Abstract

Health issues associated with gout and increased occurrence of osteoporosis or fractures have been raised; however, the results are elusive. Herein, we explored the possible link between gout and incident osteoporosis/osteoporotic fractures based on long-term follow-up nationwide data. This study enrolled 16,305 patients with gout and 65,220 controls who were matched by propensity score at a 1:4 ratio on the basis of sex, age, income, and residence from the Korean National Health Insurance Service-Health Screening Cohort database (2002–2015). A Cox proportional hazard model was employed to identify the relevance between gout and incident osteoporosis/fractures, following adjustment for various covariates. In the follow-up period, osteoporosis developed in 761 individuals with gout and 2805 controls (incidence rates: 8.0 and 7.3/1000 person-years, respectively), and each osteoporotic fracture in the distal radius (2.8 vs. 2.7/1000 person-years), hip (1.3 vs. 1.3/1000 person-years), and spine (4.5 vs. 4.5/1000 person-years) occurred in gout and control groups, respectively. After adjustment, the gout group presented an 11% higher development of osteoporosis (95% confidence interval = 1.02–1.20) than the controls (*p* = 0.011). Subgroup analyses maintained the augment of incident osteoporosis in sufferers with gout, particularly in either men or <60 years. However, no such relevance was identified between gout and incident osteoporotic fractures at any site. In conclusion, gout may result in a slightly elevated likelihood of developing osteoporosis, and not osteoporotic fractures, in the Korean population.

## 1. Introduction

Gout represents the commonly encountered inflammatory arthritis associated with hyperuricemia, causing to deposit of monosodium urate crystals within the joints, most often in the first metatarsal joint of the foot [1]. Recurrent severe painful gout attacks lasting several days or weeks may create progressive disability and further aggravate a personal quality of life [2]. This disease increases with age, with a peak at the age of 80, with 2.9–4.5-fold more male preponderance [3,4]. Recently, the prevalence of gout has been rising worldwide, with an expansion in the aging population and metabolic disorders [5]. In Korea, gout showed a 2.01% prevalence rate in 2015 [6], which constitutes a 5.15-fold enlargement compared to 0.39% in 2002, with a parallel yearly average growth of 10.8% in health insurance expenses due to gout [6]. This augment is more substantial than that observed in other countries; the prevalence of gout built up 1.64-times in the UK between 1997 and 2012 [5] and 1.4-fold in the USA from 1988 to 1994 and 2007 to 2008 [3], and 1.12-fold in Taiwan between 2005 and 2010 [4,7]. Considering Korea’s aging society and lifestyle changes over several decades, gout and its accompanying diseases have become recognized as significant public health concerns [6].

With strong associations between gout and various comorbidities [1,8,9], special attention has been paid to globally repetitive cohort studies that have documented the potential link of gout with the hazard of osteoporosis or osteoporotic fractures [10,11]. Osteoporosis primarily affects the elderly, is described as lowered bone density and enhanced bone fragility, and puts patients at peril for fractures [12,13]. It has been hypothesized that monosodium urate crystal-induced inflammation may cause a harmful outcome on bones by stimulating a cascade of complex inflammatory signaling pathways and pro-inflammatory molecules [14]. In vitro studies have shown that the urate crystal-induced inflammatory state induces rapidly create interleukin (IL)-1 to increase IL-6 and tumor necrosis factor-α (TNF-α) to evoke excessive osteoclast formation and blocks osteoblast activity [14,15], which may suppress bone formation and accelerate the resorption of bone, hence elevating the risk of fractures. Cohort studies based in Taiwan and China have indicated that women with gout have an increased osteoporotic fracture risk [10,11], especially in those harboring either a high level of total cholesterol or osteoporosis; however, no such association was found in men [11]. Not considering lifestyle-related factors [10] or a relatively small cohort [11] may have confounded those findings. One prospective cohort study utilizing the Nurses’ Health data in the USA could confirm the repercussion of gout on the hazard of incident hip fractures in women from 14 years of follow-up [16]; however, the lack of male data restricts the interpretation of the results. Moreover, another population study in Denmark emphasized the potential for osteoporotic fractures in patients with gout, regardless of sex [17].

On the other hand, conflicting conclusions have existed about the relevance of gout to the occurrence of osteoporosis or fractures. One cohort study founded on the USA commercial health plan (2004–2013) with a 2-year follow-up did not find any elevated risk of wrist and hip fractures among patients with gout [18]. Likewise, an extensive primary care medical data-based study in the United Kingdom reported no impact of gout on vertebral or non-vertebral fractures [19]. A meta-analysis concluded no definite identifiable risk for osteoporotic fractures at any site following gout in either males or females [20]. While most epidemiological studies have brought into focus the possible hazard for osteoporotic fractures following gout [11,16,17,18,19,20], data on incident osteoporosis that can increase fracture susceptibility are scarce [21,22]. Fewer than ten studies have been published on gout and the subsequent development of osteoporosis [21,22] or fracture [11,16,17,18,19,20] since 2016, of which only two cohort studies dealt with osteoporosis risk [21,22], which has yet to be reproduced in combined analyses with both osteoporosis and vertebral and non-vertebral osteoporotic fractures in enough longstanding follow-up data. Therefore, further investigation is needed to validate the suspicious relevance of gout to subsequent osteoporosis or fracture using nationwide cohort data with enough follow-up periods.

Herein, we hypothesized that individuals with gout might have increased odds of developing osteoporosis or osteoporotic fractures. This study extended the former studies in the field; we adjusted to balance the baseline characteristics between patient and control cohorts well and browsed over a 13-year long-term follow-up using the well-organized nationwide health care database.

## 2. Materials and Methods

### 2.1. Study Design and Participant Selection

The Ethics Committee of Hallym University permitted the current study (2019-10-023) and exempted the requirement for written informed consent. This study was conducted to conform to the regulations of the Ethics Committee of Hallym University. The present study made use of the Korean National Health Insurance Service-Health Screening Cohort (KNHIS-HSC) database, which furnishes Korean population-based and longitudinal information for research aims using an arbitrary sampling manner. The KNHIS runs an obligatory national health insurance system in the Republic of Korea that has registered over 98% of all Korean citizens since 1999. The KNHIS-HSC details have been formerly delineated [23]. The available data and information out of the KNHIS-HSC were entirely anonymous and de-identified. The diagnostic codes employed in the KNHIS-HSC data comply with the International Classification of Diseases, Tenth Revision, Clinical Modification (ICD-10-CM).

We retrospectively designed a longitudinal follow-up study based on a gout group and a control group without a gout history to estimate the effect of gout on the odds of incident osteoporosis or fractures. In the beginning, the members of gout, defined as ICD-10 code M10 (gout), were initially browsed out of 514,866 participants aged ≥ 40 with 615,488,428 medical claim codes from at least two clinic visits from 2002 to 2015 (*n* = 20,739). Participants who were diagnosed with osteoporosis in 2002 (1-year wash-out period, *n* = 2451) did not have documents of blood pressure level (*n* = 1) or had been diagnosed with osteoporosis/osteoporotic fractures (hip fracture, distal radius fracture, or spine fracture) before their diagnosis of gout (*n* = 1982) were excluded.

Participants that were enrolled in the control group did not correspond to the gout group between 2002 and 2015 (*n* = 494,127). People in the control group were excluded if they had any missing records since 2003 (*n* = 34) or if they had been assigned the ICD-10 code M10 (gout) once (*n* = 10,221).

**Figure 1:**
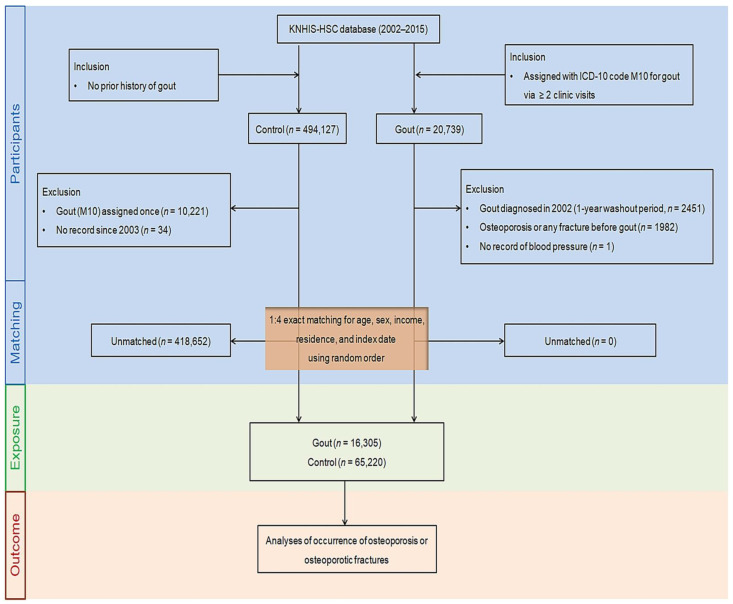
A summary chart illustrating the participant selection process used in the present study. Finally, 16,305 patients with gout were matched with 65,220 control people for age, sex, income, and region of residence out of 514,866 individuals in the Korean National Health Insurance Service-Health Screening Cohort (KNHIS-HSC) database. Abbreviations: KNHIS-HSC, the Korean National Health Insurance Service-Health Screening Cohort; ICD-10, the International Classification of Diseases, Tenth Revision, Clinical Modification.

Propensity score matching was performed at a ratio of 1:4 for sex, age, economic level, and residential region to minimize heterogeneity in the baseline demographic and clinical attributes of gout and comparison groups. To reduce selection bias, non-gout members were adopted randomly in number order. The respective index date of every participant with gout was appointed along the day when the diagnostic code of gout (M10) was electronically set aside in the health insurance claims database. Each index date of the non-gout members was determined as that of the matched counterparts with gout. Thus, the matched gout and control groups had the corresponding index dates. Any individuals in the comparison group who died or were diagnosed with osteoporosis/osteoporotic fractures prior to the index date were also discounted. Consequently, 418,652 control participants were eliminated in the process of the matching. A total of 16,305 patients with gout were matched with 65,220 control people (Figure 1).

Subsequently, we reviewed the outbreak of newly diagnosed osteoporosis or osteoporotic fractures (newly assigned ICD-10 codes for osteoporosis or osteoporotic fractures) in gout and comparison groups from the respective person’s index date until the last day of 2015.

### 2.2. Definition of Gout (Independent Variable)

The term gout in the study was stipulated as gout diagnosed or treated ≥ 2 times using ICD-10 codes (M10) [24].

### 2.3. Definition of Osteoporosis and Osteoporotic Fractures (Dependent Variable)

Osteoporosis was designated as ICD-10 codes M80 (osteoporosis with pathological fracture), M81 (osteoporosis without pathological fracture), and M82 (osteoporosis in diseases classified elsewhere) [25,26]. Amongst them, we chose only people who underwent more than two times treatments in basis upon the bone density test of X-ray or computed tomography (Claim code: HC341-HC345, E7001-E7004) [25,26].

Osteoporotic fractures on the hip, distal radius, and spine other than the cervical region were included [27], whereas cervical spinal fractures were not included and were deemed not to be affected by osteoporotic fractures [27]. Hip fracture was indicated as a fracture of the head and neck of the femur (S720), pertrochanteric fracture (S721), or subtrochanteric fracture of the femur (S722) [28]. Distal radius fracture was determined as a fracture of the lower end of the radius (S525) [29]. Spinal fractures were designated as fractures of the thoracic vertebrae (S220) or lumbar vertebrae (S320).

### 2.4. Covariates

The age distribution was categorized into ten subgroups with 5-year gaps. Economic income level was divided into five classes (class 1 (lowest income) to 5 (highest income)). Residential areas were classified into urban and rural regions [30]. Alcohol drinking, tobacco smoking, and obesity status using body mass index (BMI) were grouped equivalently [31]. The systolic blood pressure (SBP, mmHg), total cholesterol (mg/dL), diastolic blood pressure (DBP, mmHg), and fasting blood glucose (mg/dL) were quantified. The Charlson Comorbidity Index (CCI), broadly exploited to weigh disease burden using 17 disorders, was calculated to evaluate the comorbidities of individuals from 0 (no comorbidities) to 29 (multiple comorbidities) [32].

### 2.5. Statistical Analyses

The overall features of the cohort groups were compared by calculating the standardized differences. Propensity scores were computed using multivariable logistic regression on baseline covariates, including age, sex, income, and region of residence [33]. Propensity score matching was applied to curtail the effect of probable confounding elements and selection bias to keep heterogeneity between two cohort groups to a minimum. In this step, participants with gout undertook to match with comparison people according to similar propensity score values [33]. To reduce the possibility of intergroup bias, we surveyed the balance status of the matched data between groups for absolute standardized differences in covariates before and after reaching. A fundamental standardized difference of <0.20 implied an appropriate balance in terms of a specific covariate between groups [34]. Imbalance covariates with an absolute standardized difference > 0.20 after matching were further rectified via the Cox proportional hazard model [34].

Kaplan–Meier analysis and the log-rank test were employed to analyze the cumulative probability of incident osteoporosis/osteoporotic fractures in the gout group compared to the counterpart group. A stratified Cox proportional hazard model was exploited to investigate the hazard ratios (HRs) with 95% confidence intervals (CIs) for osteoporosis/osteoporotic fractures in patients with gout compared to those in control participants. In this analysis, the crude and adjusted models were used (for osteoporosis, the model was adjusted for obesity, CCI score, smoking status, alcohol consumption, total cholesterol, SBP, DBP, and fasting blood glucose; for osteoporotic fractures, the model was adjusted for the above variables plus osteoporosis). The analyses were stratified for matched variables given sex, age, income, and region of residence.

Subgroup analyses were carried out based on age (<60 years old and ≥60 years old) and sex (male and female) and discovered in the crude and adjusted models.

Two-tailed analyses were conducted, and statistical significance was determined as *p* < 0.05. The Bonferroni correction was applied for multiple comparisons. SAS version 9.4 (SAS Institute Inc., Cary, NC, USA) was run for statistical analyses.

## 3. Results

### 3.1. Baseline Characteristics

The baseline characteristics of gout and control groups after propensity score matching are outlined in Table 1. As a whole, 16,305 participants with gout and 65,220 matched controls were enlisted in the analysis of 20,739 newly diagnosed patients with gout between 2002 and 2015. The demographics of sex, age, economic level, and residence were equivalent between the two groups (standardized difference = 0). A balance of covariates was attained between the groups (standardized difference ≤ 0.2), except for obesity, as the gout group had a marginally more significant proportion of participants with obesity than the comparison group (46.7% vs. 34.6%).

### 3.2. The Occurrence of Osteoporosis in the Gout and Control Groups

**Figure 2:**
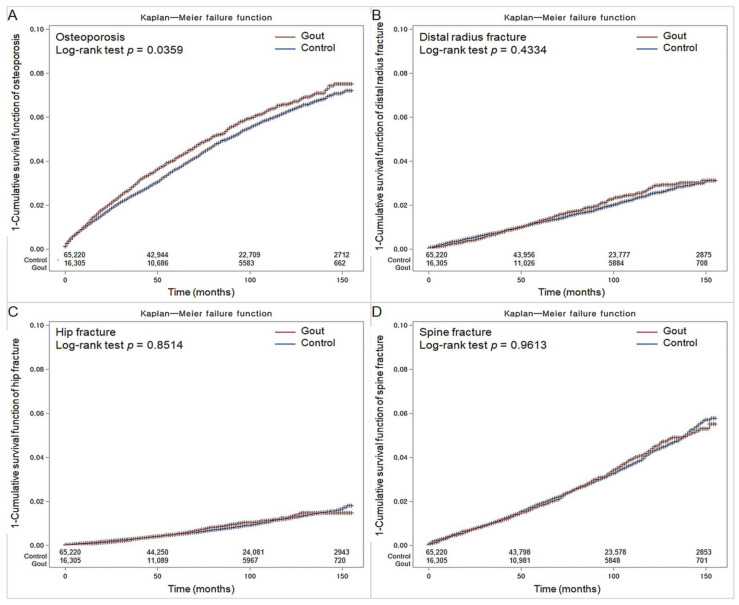
Kaplan-Meyer incidence probability of osteoporosis (**A**), distal radius fracture (**B**), hip fracture (**C**), and spine fracture (**D**) in patients with gout and control populations within 13 years of the index date.

Osteoporosis occurred in 761 (4.7%) participants in the gout group (*n* = 16,305) and 2805 (4.3%) in the control group (*n* = 65,220). Over 13 years (between 2002 and 2015, the cohort accumulated 94,898 person-years in the gout group and 382,132 person-years in the comparison group. Overall, the incidence rates of osteoporosis were 8.0 and 7.3 per 1000 person-years in gout and non-gout groups, respectively. The Kaplan–Meier analysis and log-rank test discovered a higher probability of incident osteoporosis in participants who suffered from gout than in the non-gout group participants (*p* = 0.0359; Figure 2A).

Cox proportional hazard analysis disclosed that people with gout carried a more increased likelihood of subsequent osteoporosis than those in the control group in the crude (HR 1.10; 95% CI = 1.01–1.19; *p* = 0.022) and adjusted models (HR 1.11; 95% CI = 1.02–1.20; *p* = 0.011) at the 13-year-follow-up, even after adjusting for demographic and lifestyle factors and medical comorbidities (Table 2).

As sex and age are related to the development of both gout and osteoporosis, we additionally categorized the cohorts on the basis of sex and age to identify the possible connection between gout and subsequent osteoporosis. In stratification, the hazard of subsequent osteoporosis was significantly elevated in the gout group among patients who were <60 years old and were male ((HR 1.22; 95% CI = 1.06–1.41; *p* = 0.005) and (HR 1.13; 95% CI = 1.001.28; *p* = 0.043), respectively).

### 3.3. The Occurrence of Osteoporotic Fractures

The incidence and likelihood of osteoporotic fractures in the distal radius, hip, and spine were not statistically different between gout and control groups (all *p* > 0.05; Table 3, Table 4 and Table 5). During follow-up, osteoporotic fractures of the distal radius (277 (1.7%) vs. 1052 (1.6%)), hip (127 (0.78%) vs. 500 (0.77%)), and spine (437 (2.7%) vs. 1746 (2.7%)) occurred in patients with gout and the controls.

Similar probabilities of developing osteoporotic fractures on the distal radius, hip, or spine were observed between gout and control groups in the Kaplan–Meier analysis (*p* = 0.4334, *p* = 0.8514, and *p* = 0.9613, respectively; Figure 2B–D).

The crude and adjusted HRs for osteoporotic fractures in the gout cohort were not significantly more powerful compared to those in the control group (for the distal radius, (HR, 1.06; 95% CI = 0.93–1.21; *p* = 0.401) and (HR 1.06; 95% CI = 0.93–1.21; *p* = 0.413), respectively; for the hip, (HR 1.02; 95% CI = 0.84–1.24; *p* = 0.872) and (HR 1.00; 95% CI = 0.82–1.22; *p* = 0.997), respectively; for the spine ((HR 1.00; 95% CI = 0.90–1.12; *p* = 0.943) and (HR 0.98; 95% CI = 0.88–1.09; *p* = 0.642), respectively).

Likewise, subgroup analyses through age and sex stratifications also indicated no meaningful associations for the subsequent osteoporotic fractures in patients with gout compared to the controls.

## 4. Discussion

Throughout nationwide cohort data, this study indicated that patients with gout in the Korean adult population exhibited a modest increase in the incidence of subsequent osteoporosis at the 13-year follow-up. However, no such association has been observed with osteoporotic fractures. Our results may indicate the potential likelihood of the occurrence of osteoporosis during the disease course of gout, which may further require information and education for incident osteoporosis as gout-related comorbidity.

Despite the rapidly increasing prevalence and substantial public health impact of gout, there has been a paucity of large-scale population-based information on whether gout may affect the occurrence of osteoporosis. Only two studies have been discussed in the past few years [21,22]. In a large Taiwanese longitudinal follow-up study recruiting 36,458 participants with gout and 71,602 controls, the gout cohort illustrated a 1.2 times more significant risk of osteoporosis (95% CI = 1.06–1.35) than the control cohort [22]. In another small Turkish case–control study that enrolled 75 patients who suffered from gout and 55 controls, the occurrence of subsequent osteoporosis was more boosted in the gout group than in the healthy counterpart cohort. Moreover, there were significantly low osteocalcin (a bone formation marker) levels in each group with either gout or osteoporosis, suggesting an inferior turnover rate forming new bone in patients with gout as low as those of osteoporosis [21]. Our findings accord with the former studies that proposed a correlation between gout and an increased risk of osteoporosis [21,22]. Overall, we found a slightly increased incidence of osteoporosis among participants with gout compared to those without gout (8.0 and 7.3 per 1000 person-years). This difference was confirmed to be statistically significant in those patients with gout displayed an 11% bigger likelihood (95% CI = 1.02–1.20) of occurring osteoporosis than those without gout via the Cox proportional hazard analysis with wide adjustment for confounding factors, including sex, age, socioeconomic status, SBP or DBP, total cholesterol, fasting blood glucose, obesity, smoking, alcohol consumption, and comorbidities. The present study revealed that gout per se may be an independent risk factor for osteoporosis over a 13-year follow-up period. Notably, the statistical significance of the effect of gout on incident osteoporosis appears to be identified only in long-term follow-up studies for at least 8 years [10,17,22], suggesting that it may be demanding to explore the risk of osteoporosis in patients with gout owing to the necessity for enough long-period follow-up.

When stratifying according to age and sex, we noted an increased probability of incident osteoporosis in either men or those < 60 years among the gout cohort. These results indicated that men and women with gout have different hazards for the likelihood of osteoporosis, with a 13% increase for male patients (95% CI = 1.00–1.28) and borderline significance for women (HR 1.11; 95% CI = 1.00–1.24, *p* = 0.055). This result is highly compatible with a previous report referring to distinct levels of osteoporosis risk according to sex, with a 33% increased risk for men (95% CI = 1.10–1.61) and a statistically unreached association for women (HR 1.11; 95% CI = 0.95–1.30, *p* = 0.18) [22]. In the present study, patients with gout < 60 years old harbored a 22% increased likelihood (95% CI = 1.06–1.41) of developing osteoporosis compared to those without gout. When Kok et al. [22] stratified age subgroups in more detail, they also found a moderate elevation of osteoporosis risk in two age bands of 20–39 years old (HR 1.90; 95% CI = 1.01–3.58) and ≥80 years old (HR 1.66; 95% CI = 1.12–2.48) among the gout cohort in comparison to the counterpart group. As Korea had a much lower prevalence of gout (2.01%) in 2015 [6] than 6.25% in Taiwan in 2010, one of the highest worldwide [7], the effect size of gout impacting the likelihood of developing osteoporosis relevant to age may be less likely to reach a perfect agreement. Therefore, clinical risk evaluation of osteoporosis in a subset of individuals with gout may be warranted in the Korean population, especially in men < 60 years.

In the present investigation, we found no specific associations of osteoporotic fractures in the distal radius, hip, and spine in patients with gout in comparison with those without gout. Similarly, a meta-analysis founded upon seven qualified research has shown that gout is less likely to elevate the hazard of fractures irrespective of fracture site or sex [20], indicating an unclear causal relationship between fracture and gout. Another study that conjugated the national health insurance information in Taiwan, a country with a health insurance policy close to that of Korea, noted that the gout cohort bore a statistically non-significant limited augment in the hazard for spine fractures compared to that of the control group (1.03; 95% CI = 0.70–1.51, *p* = 0.89) [10]. Instead, the risk of non-osteoporotic fractures in the extremities and spine increased in the gout cohort [10]. A recent cross-sectional study performed at a single institution in Spain revealed that patients with gout that had cardiovascular events exhibited a 5.21-fold enhanced risk of osteoporotic spine fractures (95% CI = 1.32–20.61) [35], implying that gout alone does not appear to independently affect fracture outcomes. Similarly, drug intervention lowering serum hyperuricemia did not decline the chance of fracture in the gout group [19], suggesting that a single factor such as hyperuricemia is less likely to determine fracture events in patients with gout [18,19].

The possible pathophysiological associations of gout leading to incident osteoporosis are elusive and may involve genetic, lifestyle, and environmental factors and other undefined vital factors. Recently, a large genome-wide association study demonstrated that significant genetic variants related to urate are positively associated with bone mineral density, where some genes overlap between men and women [36]. These findings highlight the presence of genetic susceptibility to osteoporosis in patients with gout depending on sex [36]. Gout and osteoporosis appear to overlap for some risk factors, including increased age, postmenopausal women, comorbidities, smoking, low physical activity, alcohol drinking, hyperglycemia, and high triglyceride level [4,22,37]. This may confer to systemic metabolic shifts escorted by increased oxidative stress and unrelenting inflammatory states, making susceptible adults more prone to the leading mechanisms of gout and osteoporosis [38]. Gouty arthritis is an inflammatory disease [15]. Specific clinical and molecular evidence advocates that inflammation greatly influences bone turnover and osteoporosis induction [13,39,40]. Numerous pro-inflammatory cytokines have been involved in the control of osteoblasts and osteoclasts, and a switch to an active immune profile has been hypothesized to be one of the essential risk factors [13]. Thus, specific pro-inflammatory molecules and immune system remodeling that overlap between gout and osteoporosis may be candidate pathogenic factors for the temporal link between these two diseases. For example, IL-1, IL-6, and TNF-α belong to the critical pro-inflammatory cytokines involved in osteoporosis [39,41] and gout [15,42]. The deposition and phagocytosis of monosodium urate crystals in tissues trigger a robust inflammatory response, mainly recruiting neutrophils and promoting IL-1ß and TNF-α production from monocytes under co-stimulation with free fatty acids or lipopolysaccharides, inducing the active central signaling of the cytoplasmic NOD-like receptor family pyrin domain containing 3 (NLRP3) inflammasome with a sustained release of IL-1ß, which leads to the induction of inflammatory caspases (e.g., IL-6 and IL-8) and propagation of inflammasome assembly and activation [15,41,42,43]. IL-1 is a powerful provoker of bone resorption [41] and has been connected with escalated bone loss in idiopathic and postmenopausal osteoporosis [44]. IL-6 and TNF-α are also pro-osteoclastic molecules that are markedly increased in patients with osteoporosis [13,45].

The integrity of this study is first based on representative, nationwide population data widely adjusted for socioeconomic status (e.g., income and area of residence), possible lifestyle-related risk elements (e.g., alcohol, blood pressure, obesity, fasting blood glucose or total cholesterol level, and smoking), and comorbidities. Second, a well-balanced study and control participants through propensity score matching may have supported our study, which may minimize selection bias and mimic randomized trials. Although gout is highly prevalent in men and at older ages, the large number of 16,305 individuals with gout could be ideally and uniformly matched to the relevant 65,220 non-gout participants in the respective age groups, which achieved balanced sex and age distributions. Third, since the KNHIS-HSC data includes every hospital and clinic across the nation without exception, complete medical histories could be obtained in the follow-up duration, allowing the generalizability and reliability of the research findings. At last, long-term follow-up may be an additional advantage. This study represents one of the most considerable nationwide follow-up analyses over 13 years for the relation of gout with the likelihood of osteoporosis.

Our study carried a couple of limitations that need to be addressed. First, as this study registered patients based on diagnosis codes and contained only Korean citizens, the influence of unmeasured confounders could not be discounted entirely. Second, no information pertinent to family history, personal genetic data, or a diet for gout or osteoporosis was unavailable in the KNHIS-HSC database. Accordingly, missing data were not considered.

## 5. Conclusions

In summary, our results may cautiously inform the modestly increased likelihood of incident osteoporosis in a subset of patients with gout during a long-term follow-up, especially in either men or those under 60; however, no such association with osteoporotic fractures was identified. Therefore, patients with gout may require screening for the early detection and appropriate treatment of osteoporosis as an avenue to manage gout.

## Figures and Tables

**Table 1:** Baseline characteristics of participants of the gout group and control group.

Characteristics	Total Participants		
	Gout (*n* = 16,305)	Control (*n* = 65,220)	Standardized Difference
Age (years old, *n*, %)			0.00
40–44	571 (3.5)	2284 (3.5)	
45–49	2008 (12.3)	8032 (12.3)	
50–54	3309 (20.3)	13,236 (20.3)	
55–59	3101 (19.0)	12,404 (19.0)	
60–64	2531 (15.5)	10,124 (15.5)	
65–69	2134 (13.1)	8536 (13.1)	
70–74	1488 (9.1)	5952 (9.1)	
75–79	823 (5.1)	3292 (5.1)	
80–84	277 (1.7)	1108 (1.7)	
≥85	63 (0.4)	252 (0.4)	
Sex (*n*, %)			0.00
Male	13,941 (85.5)	55,764 (85.5)	
Female	2364 (14.5)	9456 (14.5)	
Income (*n*, %)			0.00
1 (lowest)	2175 (13.3)	8700 (13.3)	
2	1997 (12.3)	7988 (12.3)	
3	2500 (15.3)	10,000 (15.3)	
4	3430 (21.0)	13,720 (21.0)	
5 (highest)	6203 (38.0)	24,812 (38.0)	
Region of residence (*n*, %)			0.00
Urban	7048 (43.2)	28,192 (43.2)	
Rural	9257 (56.8)	37,028 (56.8)	
Total cholesterol level (mg/dL, mean, SD)	200.2 (40.1)	196.1 (37.1)	0.11
SBP (mmHg, mean, SD)	130.2 (17.3)	127.4 (16.4)	0.17
DBP (mmHg, mean, SD)	81.1 (11.2)	79.3 (10.7)	0.16
Fasting blood glucose level (mg/dL, mean, SD)	101.6 (28.8)	102.1 (30.9)	0.02
Obesity ^†^ (*n*, %)			0.29
Underweight	193 (1.2)	1510 (2.3)	
Normal	3999 (24.5)	22,726 (34.9)	
Overweight	4507 (27.6)	18,411 (28.2)	
Obese I	6945 (42.6)	21,068 (32.3)	
Obese II	661 (4.1)	1505 (2.3)	
Smoking status (*n*, %)			0.08
Nonsmoker	8886 (54.5)	34,681 (53.2)	
Past smoker	3403 (20.9)	12,280 (18.8)	
Current smoker	4016 (24.6)	18,259 (28.0)	
Alcohol consumption (*n*, %)			0.11
<1 time a week	8108 (49.7)	35,919 (55.1)	
≥1 time a week	8197 (50.3)	29,301 (44.9)	
CCI score (score, *n*, %)			0.13
0	10,359 (63.5)	45,309 (69.5)	
1	2425 (14.9)	8563 (13.1)	
2	1490 (9.1)	4971 (7.6)	
≥3	2031 (12.5)	6377 (9.8)	
Osteoporosis (*n*, %)	761 (4.7)	2805 (4.3)	0.02
Distal radius fracture (*n*, %)	277 (1.7)	1052 (1.6)	0.01
Hip fracture (*n*, %)	127 (0.8)	500 (0.8)	0.00
Spine fracture (*n*, %)	437 (2.7)	1746 (2.7)	0.00

Abbreviations: CCI, Charlson comorbidity index; DBP, diastolic blood pressure; SBP, systolic blood pressure; SD, standard deviation. ^†^ Obesity (BMI, body mass index, kg/m^2^) was categorized as <18.5 (underweight), ≥18.5 to <23 (normal), ≥23 to <25 (overweight), ≥25 to <30 (obese I), and ≥30 (obese II).

**Table 2:** Hazard ratio (95% confidence interval) for osteoporosis in the gout and control groups with subgroup analyses according to age and sex.

Characteristics	No. of Osteoporosis/ No. of Participants	Follow-Up Duration, Person-Years	Incidence Rate, Per 1000 Person-Years	Hazard Ratios for Osteoporosis			
				Crude ^†^	*p*-Value	Adjusted ^‡^	*p*-Value
Total participants (*n* = 81,525)							
Gout	761/16,305	94,898	8.0	1.10 (1.01–1.19)	0.022	1.11 (1.02–1.20)	0.011 *
Control	2805/65,220	382,132	7.3	1		1	
Age < 60 years old (*n* = 44,945)							
Gout	255/8989	60,212	4.2	1.21 (1.05–1.39)	0.009 *	1.22 (1.06–1.41)	0.005 *
Control	859/35,956	242,560	3.5	1		1	
Age ≥ 60 years old (*n* = 36,580)							
Gout	506/7316	34,686	14.6	1.05 (0.95–1.16)	0.324	1.06 (0.96–1.17)	0.250
Control	1946/29,264	139,572	13.9	1		1	
Males (*n* = 69,705)							
Gout	344/13,941	82,815	4.2	1.08 (0.96–1.22)	0.189	1.13 (1.00–1.28)	0.043
Control	1270/55,764	332,450	3.8	1		1	
Females (*n* = 11,820)							
Gout	417/2364	12,083	34.5	1.11 (1.00–1.24)	0.058	1.11 (1.00–1.24)	0.055
Control	1535/9456	49,682	30.9	1		1	

* Stratified Cox proportional hazard model, Significance at *p* < 0.05 with Bonferroni correction. ^†^ Models were stratified by age, sex, income, and region of residence. ^‡^ Adjusted for total cholesterol, systolic blood pressure, diastolic blood pressure, fasting blood glucose, obesity, smoking, alcohol consumption, and Charlson comorbidity index score.

**Table 3:** Hazard ratio (95% confidence interval) for distal radius fracture in the gout and control groups with subgroup analyses according to age and sex.

Characteristics	No. of Distal Radius Fracture/ No. of Participants	Follow-Up Duration, Person-Years	Incidence Rate, Per 1000 Person-Years	Hazard Ratios for Distal Radius Fracture			
				Crude ^†^	*p*	Adjusted ^‡^	*p*
Total participants (*n* = 81,525)							
Gout	277/16,305	97,976	2.8	1.06 (0.93–1.21)	0.401	1.06 (0.93–1.21)	0.413
Control	1052/65,220	392,452	2.7	1		1	
Age < 60 years old (*n* = 44,945)							
Gout	142/8989	61,058	2.3	1.09 (0.91–1.31)	0.367	1.08 (0.90–1.31)	0.411
Control	525/35,956	245,087	2.1	1		1	
Age ≥ 60 years old (*n* = 36,580)							
Gout	135/7316	36,918	3.7	1.03 (0.85–1.24)	0.774	1.03 (0.85–1.25)	0.766
Control	527/29,264	147,365	3.6	1		1	
Males (*n* = 69,705)							
Gout	169/13,941	83,807	2.0	1.04 (0.88–1.23)	0.666	1.06 (0.89–1.26)	0.519
Control	652/55,764	335,341	1.9	1		1	
Females (*n* = 11,820)							
Gout	108/2364	14,169	7.6	1.09 (0.88–1.35)	0.414	1.05 (0.85–1.31)	0.645
Control	400/9,456	57,111	7.0	1		1	

Stratified Cox proportional hazard model, Significance at *p* < 0.05 with Bonferroni correction. ^†^ Models were stratified by age, sex, income, and region of residence. ^‡^ Adjusted for total cholesterol, systolic blood pressure, diastolic blood pressure, fasting blood glucose, obesity, smoking, alcohol consumption, osteoporosis history and Charlson comorbidity index score.

**Table 4:** Hazard ratio (95% confidence interval) for hip fracture in the gout and control groups with subgroup analyses according to age and sex.

Characteristics	No. of Hip Fracture/ No. of Participants	Follow-Up Duration, Person-Years	Incidence Rate, Per 1000 Person-Years	Hazard Ratios for Hip Fracture			
				Crude ^†^	*p*	Adjusted ^‡^	*p*
Total participants (*n* = 81,525)							
Gout	127/16,305	98,689	1.3	1.02 (0.84–1.24)	0.872	1.00 (0.82–1.22)	0.997
Control	500/65,220	395,473	1.3	1		1	
Age < 60 years old (*n* = 44,945)							
Gout	31/8989	61,573	0.5	1.27 (0.85–1.90)	0.245	1.23 (0.81–1.85)	0.335
Control	98/35,956	247,060	0.4	1		1	
Age ≥ 60 years old (*n* = 36,580)							
Gout	96/7316	37,116	2.6	0.95 (0.76–1.19)	0.679	0.94 (0.75–1.18)	0.604
Control	402/29,264	148,413	2.7	1		1	
Males (*n* = 69,705)							
Gout	108/13,941	84,172	1.3	1.05 (0.85–1.30)	0.641	1.09 (0.88–1.35)	0.445
Control	410/55,764	336,974	1.2	1		1	
Females (*n* = 11,820)							
Gout	19/2364	14,517	1.3	0.85 (0.52–1.40)	0.527	0.71 (0.42–1.19)	0.191
Control	90/9456	58,499	1.5	1		1	

Stratified Cox proportional hazard model, Significance at *p* < 0.05 with Bonferroni correction. ^†^ Models were stratified by age, sex, income, and region of residence. ^‡^ Adjusted for total cholesterol, systolic blood pressure, diastolic blood pressure, fasting blood glucose, obesity, smoking, alcohol consumption, osteoporosis history and Charlson comorbidity index score.

**Table 5:** Hazard ratio (95% confidence interval) for spine fracture in the gout and control groups with subgroup analyses according to age and sex.

Characteristics	No. of Spine Fracture/ No. of Participants	Follow-Up Duration, Person-Years	Incidence Rate, Per 1000 Person-Years	Hazard Ratios for Spine Fracture			
				Crude ^†^	*p*	Adjusted ^‡^	*p*
Total participants (*n* = 81,525)							
Gout	437/16,305	97,555	4.5	1.00 (0.90–1.12)	0.943	0.98 (0.88–1.09)	0.642
Control	1746/65,220	390,661	4.5	1		1	
Age < 60 years old (*n* = 44,945)							
Gout	132/8989	61,194	2.2	1.11 (0.92–1.35)	0.287	1.06 (0.87–1.29)	0.562
Control	479/35,956	245,546	2.0	1		1	
Age ≥ 60 years old (*n* = 36,580)							
Gout	305/7316	36,361	8.4	0.96 (0.85–1.09)	0.567	0.94 (0.83–1.07)	0.359
Control	1267/29,264	145,115	8.7	1		1	
Males (*n* = 69,705)							
Gout	330/13,941	83,344	4.0	1.02 (0.91–1.16)	0.711	1.03 (0.91–1.16)	0.679
Control	1286/55,764	333,657	3.9	1		1	
Females (*n* = 11,820)							
Gout	107/2364	14,211	7.5	0.95 (0.77–1.17)	0.627	0.86 (0.69–1.06)	0.160
Control	460/9456	57,004	8.1	1		1	

Stratified Cox proportional hazard model, Significance at *p* < 0.05 with Bonferroni correction. ^†^ Models were stratified by age, sex, income, and region of residence. ^‡^ Adjusted for total cholesterol, systolic blood pressure, diastolic blood pressure, fasting blood glucose, obesity, smoking, alcohol consumption, osteoporosis history and Charlson comorbidity index score.

## Data Availability

All data are available from the database of National Health Insurance Sharing Service (NHISS) https://nhiss.nhis.or.kr/ (accessed on 1 May 2022). NHISS allows access to all of these data for any researcher who promises to follow the research ethics at some processing charge. If you wish to access the data of this article, you can download them from the website after promising to follow the research ethics.
